# Real-world predictors of divorce among outpatients with bipolar disorder: sex differences and clinical implications

**DOI:** 10.1186/s12991-023-00487-6

**Published:** 2023-12-12

**Authors:** Keita Tokumitsu, Norio Sugawara, Naoto Adachi, Yukihisa Kubota, Yoichiro Watanabe, Kazuhira Miki, Takaharu Azekawa, Koji Edagawa, Eiichi Katsumoto, Seiji Hongo, Eiichiro Goto, Hitoshi Ueda, Masaki Kato, Reiji Yoshimura, Atsuo Nakagawa, Toshiaki Kikuchi, Takashi Tsuboi, Koichiro Watanabe, Norio Yasui-Furukori

**Affiliations:** 1 Department of Psychiatry, Dokkyo Medical University School of Medicine, Tochigi, Japan; 2 The Japanese Association of Neuro-Psychiatric Clinics, Tokyo, Japan; 3 The Japanese Society of Clinical Neuropsychopharmacology, Tokyo, Japan; 4 Department of Neuropsychiatry, Kansai Medical University, Osaka, Japan; 5 Department of Psychiatry, University of Occupational and Environmental Health, Fukuoka, Japan; 6 Department of Neuropsychiatry, St. Marianna University School of Medicine, Kanagawa, Japan; 7 Department of Neuropsychiatry, Keio University School of Medicine, Tokyo, Japan; 8 Department of Neuropsychiatry, Kyorin University School of Medicine, Tokyo, Japan

**Keywords:** Bipolar disorder, Divorce, Predictor, Real-world, Outpatient

## Abstract

**Background:**

Bipolar disorder is a mental illness characterized by recurring episodes of mania and depression and is known to cause social impairment. Additionally, it has been revealed that bipolar disorder increases the risk of divorce and loss of family member support, which can worsen the prognosis. However, there is limited evidence regarding the predictive factors of divorce among patients with bipolar disorder in real-world settings.

**Methods:**

This study utilized an observational approach and involved psychiatrists from 176 member clinics of the Japanese Association of Neuro-Psychiatric Clinics. They were requested to conduct a retrospective review of medical records and complete a questionnaire focused on patients diagnosed with bipolar disorder. The data collection period for baseline patient characteristics spanned from September to October 2017. Next, we investigated the incidence of divorce over a 2-year period, ranging from baseline to September to October 2019.

**Results:**

A total of 1071 outpatients with bipolar disorder were included in the analysis, and 2.8% (30/1071) experienced divorce during the first 2 years of observation. The incidence of divorce in this population was considerably higher than that in the general Japanese population. Binomial logistic regression analysis confirmed that a younger baseline age and lower BMI values were statistically significant predictors of divorce occurrence for all study participants. The predictors of divorce were then examined separately by sex. The results revealed that for men, a younger age at baseline and having bipolar I disorder compared to bipolar II disorder were statistically significant predictors of divorce. In contrast, for women, having a lower BMI and using anxiolytics emerged as statistically significant predictors of divorce.

**Conclusions:**

In this study, a younger baseline age and lower BMI values were statistically significant predictors of divorce in patients with bipolar disorder. Notably, the predictors of divorce varied significantly between men and women. These findings provide important insights from a family perspective regarding social support for individuals with bipolar disorder in real-world clinical settings.

## Background

Bipolar disorder is a chronic and severe psychiatric disorder characterized by recurrent episodes of mania and depression, affecting approximately 1–2% of the global population [1]. Bipolar disorder is classified into two subtypes: Bipolar I disorder involves severe manic episodes lasting at least 1 week, potentially leading to hospitalization, while Bipolar II disorder features milder hypomanic episodes and depressive cycles. Both subtypes impact daily functioning, with the severity of manic episodes distinguishing the two [1]. The average age of onset for bipolar I disorder is 18 years, while for bipolar II disorder, it is 20 years [2]. The prevalence of bipolar disorder is similar for both males and females [3]. Furthermore, bipolar disorder is recognized as a difficult disease to treat due to its potential comorbidity with other mental disorders, including substance use disorders, personality disorders, and developmental disorders [4]. Importantly, bipolar disorder has a profound impact on various aspects of social functioning, such as family relationships, occupational performance, and overall quality of life [4].

In addition, bipolar disorder is known to have a poorer prognosis with repeated relapses [5, 6]. Therefore, early intervention in treatment and prevention of recurrence are important therapeutic strategies. However, patients with bipolar disorder often have poor adherence, so they need a good doctor‒patient relationship and support from their families [7].

From a family context, the role of a spouse in providing social support to a patient with mental illness is substantial [8]. By offering emotional and practical support, the spouse contributes to the overall well-being and recovery of the patient. However, not only do family relationships affect the course of bipolar disorder, but it has also been reported that bipolar disorder has a strong impact on family functioning, caregiver burden and their health condition [9]. Therefore, treatment and support programs focusing on the families and caregivers of patients with bipolar disorder are effective for better outcomes for patients and benefits for caregivers [9].

On the other hand, researchers and physicians have shown interest in the divorce risk among individuals with mental illness, including bipolar disorder [10, 11]. A previous study suggested that patients with bipolar disorder are two to three times more likely to divorce and separate than the general population in the United States [12, 13]. This increased risk is attributed to factors such as emotional and financial strains, difficulties maintaining interpersonal relationships, and increased caregiver burden. In addition, divorce can have important adverse effects on patients with bipolar disorder. For example, social isolation due to divorce can lead to further worsening of psychiatric symptoms, increased risk of relapse, financial difficulties, and decreased treatment adherence [8]. Therefore, identifying predictors of divorce in bipolar patients can provide valuable insight into targeted interventions to promote family relationship stability and the overall well-being of patients and their families [14].

In previous research, it was reported that married women had fewer depressive episodes over two years and lower cumulative severity of depression compared to unmarried women. It is suggested that women may be sensitive to the positive effects of social support obtained within stable marital relationships [15]. Additionally, previous research suggests a higher likelihood of unmarried status among males with bipolar I disorder, although it is unclear whether this is associated with divorce. Interestingly, while reports exist regarding sex differences in the general population regarding the association between marital status and weight [16], it is unknown whether these results can be applied to bipolar disorder patients. Considering these results from previous research, it is thought that identifying predictors of divorce with a focus on sex differences could have a positive impact on providing psychosocial support for patients.

Notably, cultural and cross-national differences play a crucial role in understanding divorce risk factors among individuals [17]. For this reason, the risk of divorce in bipolar disorder may vary by cultural context, including stigma against mental illness, social attitudes toward marriage and divorce, and access to mental health care services. Consequently, findings from research conducted in one country may not generalize to other cultural contexts, necessitating cross-cultural investigations. Nonetheless, previous studies on bipolar disorder and divorce risk have focused on patients in Western countries, and there has been a lack of research in Japan [12, 13].

In Japan, more than 90% of patients with mood disorders receive outpatient treatment, with half of them seeking care at clinics that belong to the Japanese Association of Neuro-Psychiatric Clinics (JAPC) [18]. To advance our understanding of the practical treatment of bipolar disorder in Japan, the Japanese Association of Neuro-Psychiatric Clinics and the Japanese Society of Clinical Neuropsychopharmacology conducted joint research, known as the MUlticenter treatment SUrvey on BIpolar disorder in Japanese psychiatric clinics (MUSUBI), to collect evidence from real-world settings [18–29]. Previous research from the MUSUBI study has provided valuable insights into predictors of psychiatric hospitalization, manic/hypomanic episodes, and antidepressant prescribing patterns among outpatients with bipolar disorder [18–29]. The current study aims to identify predictors of divorce among Japanese outpatients with bipolar disorder using data from a multicenter study involving 176 clinics across Japan. To identify predictors of divorce occurrence in bipolar outpatients during the 2-year observation period, baseline sociodemographic and clinical characteristics were extracted and analyzed.

By examining risk factors for divorce in the Japanese context, this study expands the real-world evidence on bipolar disorder and family relationships. This study has the potential to inform targeted interventions and support services for individuals with bipolar disorder in Japan, ultimately promoting healthier and more stable relationships. We consider that by implementing preventive interventions for patients with predictors of divorce, we can provide social support for not only the individuals themselves but also their family members.

## Methods

### Study design and participants

The current study is a retrospective survey using questionnaires conducted at 176 JAPC-affiliated psychiatric clinics.

The MUSUBI study commenced in 2016 [19], and data collection on the marital status of participants began in 2017. Therefore, we used data from September to October 2017 as the baseline demographics and characteristics of the participants. We investigated the incidence of divorce over two years, extending from baseline to September to October 2019. Patients were diagnosed with bipolar disorder based on the ICD-10 [30]. In addition, we classified bipolar I disorder and bipolar II disorder according to the DSM-5 criteria [31]. The current study included individuals who were married at baseline and attended these psychiatric clinics.

### Study procedures

Clinical psychiatrists asked participants to complete a semistructured questionnaire by conducting a retrospective personal interview. The questionnaire encompassed participant characteristics: age at study entry, age of onset, sex, height, weight, bipolar subcategories, work status, educational background, mood status at baseline, intelligence quotient (IQ), Global Assessment of Functioning (GAF) score, any prescription of psychotropic drugs, rapid cycler at baseline or not, manic symptoms during 1 year before baseline or not, psychiatric hospitalization during 1 year before baseline or not, personality disorder, developmental disorder, physical comorbidities, substance abuse, psychotic feature and suicidal ideation. Additionally, we evaluated the occurrence of divorce throughout the two years following the baseline. Patients who were bereaved of their spouse during the observation period were excluded from the analysis.

### Statistical analysis

All statistical analyses were conducted using IBM SPSS Statistics (IBM Corporation, Version 28.0.0.0) and EZR (Saitama Medical Center, Jichi Medical University, Saitama, Japan) [32]. The EZR offers a graphical user interface for R (The R Foundation for Statistical Computing, Vienna, Austria, version 4.0.3). Specifically, it is a modified version of R Commander (version 2.7–1) that integrates statistical functions commonly employed in biostatistics.

All statistical tests were two-sided with a significance level of 0.05. Demographic and clinical characteristics were analyzed using Fisher’s exact test and Student’s t test to identify differences between participants who experienced divorce and those who did not within the two years following baseline. Univariate analyses were performed to assess demographic and clinical characteristics for participants. Furthermore, the association between divorce and the combination of various factors was analyzed. All demographic and clinical characteristics related to the occurrence of divorce among the study participants were identified using binomial logistic regression with a forced entry model to avoid missing any potential associations. These independent factors included sex, body mass index (BMI), age at baseline, age of onset, bipolar subcategories (bipolar I disorder, bipolar II disorder or unclassifiable), work status at baseline, educational background, mood status at baseline, Intelligence quotient (IQ), GAF score, prescription of psychotropic drugs (mood stabilizer, antipsychotics, antidepressants, anxiolytics, hypnotics), rapid cycler at baseline, manic symptoms during 1 year before baseline or not, psychiatric hospitalization during 1 year before baseline or not, personality disorder, developmental disorder, physical comorbidities, substance abuse (alcohol abuse), psychotic feature and suicidal ideation.

The following dummy variables for each factor were incorporated in the binomial logistic regression analysis: female = 0, male = 1; unemployed = 0, employed = 1; IQ (> 85) = 0, IQ (85 or less) = 1; psychiatric comorbidity = 1, no psychiatric comorbidity = 0; personality disorder = 1, no personality disorder = 0; developmental disorder = 1, no developmental disorder = 0; physical comorbidity = 1, no physical comorbidity = 0; substance abuse (alcohol abuse) = 1, no substance abuse (no alcohol abuse) = 0; no rapid cycler = 0, rapid cycler = 1; manic symptoms during 1 year before baseline = 1, no manic symptoms during 1 year before baseline = 0; psychiatric hospitalization during 1 year before baseline = 1, no psychiatric hospitalization during 1 year before baseline = 0; psychotic feature = 1, no psychotic feature = 0; suicidal ideation = 1, no suicidal ideation = 0; antidepressant prescription = 1, no antidepressant prescription = 0; antipsychotics prescription = 1, no antipsychotics prescription = 0; anxiolytics prescription = 1, no anxiolytics prescription = 0; hypnotics prescription = 1, no hypnotics prescription = 0. Cases with missing values in the questionnaire responses were listwise excluded in the binomial logistic regression analysis.

### Ethics

This study adhered to the principles outlined in the Declaration of Helsinki and the Japanese Ethical Guidelines for Medical and Health Research Involving Human Subjects. Before the commencement of the study, the research protocol underwent review and received approval from the Institutional Review Board of the Ethics committee of JAPC (approval No. 20160822, 2017-3, 2019-5) and the Ethics Committee of Dokkyo Medical University School of Medicine (approval No. 2020-005). As this was a retrospective review of medical records, informed consent requirements were waived; nonetheless, we disclosed information regarding this research, allowing patients the option to opt out. Our team secured the necessary administrative permissions and licenses to access the data utilized in this study. The Ethics Committee of the Japanese Association of Neuro-Psychiatric Clinics imposed data-sharing restrictions due to the potential presence of identifiable or sensitive patient information. To request data, please contact the institutional review board of the ethics committee. The contact information for our ethics committee is as follows: The Institutional Review Board of the Ethics Committee of the Japanese Association of Neuro-Psychiatric Clinics; Shibuya-ku, Yoyogi 1-38-2, Tokyo Metropolis, Japan, Postal Code 151–0053, Phone + 81-3-3320-1423.

## Results

In the present study, 1071 outpatients with bipolar disorder were included in the analysis. Of these, 2.8% (30/1071) experienced divorce during the first 2 years of observation. This incidence of divorce was considerably higher than in the general Japanese population. A Bonferroni correction was applied to the 24 comparisons made, yielding a corrected significance criterion of p < 0.0021 in univariate analyses. For all study participants, univariate analysis applying the Bonferroni correction revealed that patients who had experienced divorce had a statistically significant lower age at baseline (p < 0.001) (Table 1). The average age in the group who continued to be married was 54.91 years, while the average age in the group who experienced divorce was 46.83 years.

**Table 1:** Univariate analysis of factors for divorce among all participants

Factor	Group	Marital status		Fisher’s exact test
		Married (N = 1041)	Divorced (N = 30)	p value
Sex	Female: n (%)	535 (51.4)	15 (50.0)	1.000
	Male: n (%)	506 (48.6)	15 (50.0)	
BMI at baseline (mean (SD))		23.62 (4.10)	22.39 (4.00)	0.125
Age at baseline (mean (SD))		54.91 (12.31)	46.83 (11.64)	< 0.001
Age of onset (mean (SD))		37.33 (12.35)	32.25 (9.79)	0.031
Bipolar disorder subcategories	Bipolar I disorder: n (%)	355 (34.1)	10 (33.3)	0.433
	Bipolar II disorder: n (%)	632 (60.8)	17 (56.7)	
	Unclassifiable: n (%)	53 (5.1)	3 (10.0)	
Work status at baseline employed	Unemployed: n (%)	239 (23.1)	7 (23.3)	1.000
	Employed: n (%)	795 (76.9)	23 (76.7)	
Educational background	Special support educational school: n (%)	1 (0.1)	0 (0.0)	0.526
	Junior high school: n (%)	42 (4.2)	0 (0.0)	
	High school or vocational school: n (%)	441 (43.8)	14 (50.0)	
	Junior college or technical college: n (%)	93 (9.2)	3 (10.7)	
	Master's degree or higher: n (%)	34 (3.4)	2 (7.1)	
	University: n (%)	397 (39.4)	9 (32.1)	
Mood status at baseline	Depressive state: n (%)	336 (32.4)	13 (43.3)	0.375
	Manic/hypomanic state: n (%)	76 (7.3)	2 (6.7)	
	Mixed feature: n (%)	70 (6.7)	3 (10.0)	
	Remission: n (%)	556 (53.6)	12 (40.0)	
Intelligence quotient; IQ	< 71: n (%)	7 (0.7)	0 (0.0)	1.000
	85–71: n (%)	29 (2.9)	0 (0.0)	
	> 85: n (%)	981 (96.5)	28 (100.0)	
Global assessment of functioning; GAF	81–100: n (%)	477 (45.9)	11 (36.7)	0.668
	61–80: n (%)	407 (39.1)	15 (50.0)	
	41–60: n (%)	136 (13.1)	4 (13.3)	
	1–40: n (%)	20 (1.9)	0 (0.0)	
Mood stabilizers prescription	No: n (%)	190 (18.3)	9 (30.0)	0.148
	Yes: n (%)	851 (81.7)	21 (70.0)	
Antipsychotics prescription	No: n (%)	537 (51.6)	13 (43.3)	0.459
	Yes: n (%)	504 (48.4)	17 (56.7)	
Antidepressants prescription	No: n (%)	625 (60.0)	14 (46.7)	0.185
	Yes: n (%)	416 (40.0)	16 (53.3)	
Anxiolytics prescription	No: n (%)	698 (67.1)	16 (53.3)	0.120
	Yes: n (%)	343 (32.9)	14 (46.7)	
Hypnotics prescription	No: n (%)	427 (41.0)	11 (36.7)	0.709
	Yes: n (%)	614 (59.0)	19 (63.3)	
Rapid cycler at baseline	No: n (%)	965 (92.8)	28 (93.3)	1.000
	Yes: n (%)	75 (7.2)	2 (6.7)	
Manic symptoms during 1 year before baseline	No: n (%)	733 (70.5)	23 (76.7)	0.546
	Yes: n (%)	307 (29.5)	7 (23.3)	
Hospitalization during 1 year before baseline	No: n (%)	1019 (97.9)	30 (100.0)	1.000
	Yes: n (%)	22 (2.1)	0 (0.0)	
Personality disorder	No: n (%)	994 (95.6)	25 (83.3)	0.012
	Yes: n (%)	46 (4.4)	5 (16.7)	
Developmental disorder	No: n (%)	999 (96.1)	30 (100.0)	0.625
	Yes: n (%)	41 (3.9)	0 (0.0)	
Physical comrbidity	No: n (%)	715 (68.9)	22 (73.3)	0.692
	Yes: n (%)	323 (31.1)	8 (26.7)	
Substance abuse	No: n (%)	997 (96.1)	29 (96.7)	1.000
	Yes: n (%)	41 (3.9)	1 (3.3)	
Psychotic feature	No: n (%)	992 (95.6)	28 (93.3)	0.395
	Yes: n (%)	46 (4.4)	2 (6.7)	
Suicidal ideation	No: n (%)	974 (93.8)	27 (90.0)	0.429
	Yes: n (%)	64 (6.2)	3 (10.0)	

p < 0.0021 was regarded as significant using Bonferroni’s correction due to multiple testing

Next, 122 cases with missing values in the questionnaire responses were listwise excluded because of binomial logistic regression analysis. Thus, data from 949 participants were included in the binomial logistic regression analysis. Binomial logistic regression analysis confirmed that a younger age at baseline (odds ratio = 0.941, p = 0.036) and lower BMI values (odds ratio = 0.88, p = 0.043) were statistically significant predictors of divorce occurrence for all study participants (Table 2).

**Table 2:** Binomial logistic regression analysis of factors for divorce among all participants

Variables in the equation	Coefficient	Standard error	Wald value	p value	Odds ratio (95% confidence interval)
Sex (being male)	0.952	0.513	3.438	0.064	2.590 (0.947–7.084)
BMI at baseline	−0.128	0.063	4.092	0.043	0.880 (0.777–0.996)
Age at baseline	−0.061	0.029	4.383	0.036	0.941 (0.888–0.996)
Age of onset	−0.013	0.030	0.174	0.676	0.987 (0.931–1.048)
Bipolar disorder subcategories (reference; Bipolar I disorder)			1.527	0.466	
Bipolar II disorder	−0.630	0.526	1.438	0.231	0.532 (0.190–1.492)
Unclassifiable	−0.166	0.975	0.029	0.865	0.847 (0.125–5.731)
Work status at baseline (employed)	−0.804	0.612	1.728	0.189	0.448 (0.135–1.484)
Educational background (reference; Junior college, technical college, or higher)			2.175	0.337	
Special support education school, junior high school	−16.498	5499.370	0.000	0.998	0.000 (0.000-Infinity)
High school, vocational school	0.706	0.479	2.175	0.140	2.026 (0.793–5.179)
Mood status(reference; Depressive state)			1.886	0.596	
Manic/hypomanic state	0.457	0.963	0.225	0.635	1.579 (0.239–10.423)
Mixed feature	−0.106	0.956	0.012	0.912	0.900 (0.138–5.856)
Remission	−0.719	0.629	1.310	0.252	0.487 (0.142–1.670)
Intelligence quotient (85 or less)	−17.084	5938.844	0.000	0.998	0.000 (0.000–Infinity)
Global Assessment of Functioning (reference; 81–100)			1.337	0.513	
61–80	−0.312	0.614	0.258	0.612	0.732 (0.220–2.438)
1–60	−1.154	1.008	1.311	0.252	0.315 (0.044–2.274)
Mood stabilizers prescription	−0.662	0.499	1.761	0.184	0.516 (0.194–1.371)
Antipsychotics prescription	0.399	0.484	0.679	0.410	1.490 (0.577–3.848)
Antidepressants prescription	0.578	0.505	1.312	0.252	1.782 (0.663–4.793)
Anxiolytics prescription	0.657	0.475	1.918	0.166	1.930 (0.761–4.893)
Hypnotics prescription	−0.227	0.491	0.213	0.645	0.797 (0.304–2.088)
Rapid cycler at baseline	0.433	0.937	0.214	0.644	1.542 (0.246–9.672)
Manic symptoms during 1 year before baseline	−0.410	0.697	0.346	0.557	0.664 (0.169–2.601)
Hospitalization during 1 year before baseline	−17.577	7622.567	0.000	0.998	0.000 (0.000–Infinity)
Personality disorder	1.225	0.799	2.350	0.125	3.404 (0.711–16.296)
Developmental disorder	−17.574	6004.819	0.000	0.998	0.000 (0.000–Infinity)
Physical comrbidity	0.014	0.545	0.001	0.980	1.014 (0.349–2.948)
Substance abuse	−0.516	1.223	0.178	0.673	0.597 (0.054–6.565)
Psychotic feature	−0.753	1.131	0.443	0.506	0.471 (0.051–4.324)
Suicidal ideation	0.963	0.888	1.176	0.278	2.620 (0.46–14.938)
Constant	3.445	2.093	2.710	0.100	

p < 0.05 was regarded as statistically significant using binomial logistic regression with forced entry

The predictors of divorce were then examined separately by sex. The results revealed that for men, a younger age at baseline (odds ratio = 0.896, p = 0.022) and having bipolar I disorder compared to bipolar II disorder (odds ratio of bipolar II disorder = 0.16, p = 0.016; reference factor = bipolar I disorder) were statistically significant predictors of divorce (Table 3). In contrast, for women, having a lower BMI (odds ratio = 0.671, p = 0.013) and using anxiolytics (odds ratio = 5.678, p = 0.048) were statistically significant predictors of divorce (Table 4). These results suggest that the predictors of divorce among bipolar patients are distinct for men and women.

**Table 3:** Binomial logistic regression analysis of factors for divorce among male participants

Variables in the equation	Coefficient	Standard error	Wald value	p value	Odds ratio (95% confidence interval)
BMI at baseline	0.012	0.098	0.015	0.903	1.012 (0.836–1.225)
Age at baseline	−0.109	0.048	5.249	0.022	0.896 (0.816–0.984)
Age of onset	−0.008	0.045	0.032	0.858	0.992 (0.908–1.084)
Bipolar disorder subcategories (reference; Bipolar I disorder)			5.818	0.055	
Bipolar II disorder	−1.831	0.759	5.818	0.016	0.160 (0.036–0.710)
Unclassifiable	−18.707	7560.262	0.000	0.998	0.000 (0.000-Infinity)
Work status at baseline (employed)	−0.757	0.996	0.577	0.447	0.469 (0.067–3.304)
Educational background (reference; Junior college, technical college, or higher)			2.619	0.270	
Special support education school, junior high school	−17.040	8305.015	0.000	0.998	0.000 (0.000–Infinity)
High school, vocational school	1.075	0.664	2.619	0.106	2.929 (0.797–10.765)
Mood status(reference; Depressive state)			2.856	0.414	
Manic/hypomanic state	1.361	1.299	1.097	0.295	3.899 (0.306–49.741)
Mixed feature	0.298	1.495	0.040	0.842	1.347 (0.072–25.212)
Remission	−0.935	0.903	1.073	0.300	0.393 (0.067–2.303)
Intelligence quotient (85 or less)	−16.490	9528.616	0.000	0.999	0.000 (0.000–Infinity)
Global assessment of functioning (reference; 81–100)			0.698	0.706	
61–80	−0.434	0.890	0.237	0.626	0.648 (0.113–3.707)
1–60	−1.282	1.546	0.687	0.407	0.278 (0.013–5.745)
Mood stabilizers prescription	−1.096	0.814	1.816	0.178	0.334 (0.068–1.646)
Antipsychotics prescription	0.357	0.721	0.245	0.620	1.429 (0.348–5.868)
Antidepressants prescription	1.002	0.754	1.766	0.184	2.723 (0.622–11.934)
Anxiolytics prescription	0.296	0.746	0.158	0.691	1.345 (0.312–5.804)
Hypnotics prescription	−0.290	0.702	0.171	0.679	0.748 (0.189–2.962)
Rapid cycler at baseline	0.827	1.148	0.519	0.471	2.286 (0.241–21.672)
Manic symptoms during 1 year before baseline	−0.363	1.009	0.130	0.719	0.695 (0.096–5.024)
Hospitalization	−17.619	14481.621	0.000	0.999	0.000 (0.000–Infinity)
Personality disorder	0.479	1.372	0.122	0.727	1.615 (0.110–23.749)
Developmental disorder	−17.629	7810.215	0.000	0.998	0.000 (0.000–Infinity)
Physical comrbidity	−0.273	0.840	0.106	0.745	0.761 (0.147–3.949)
Substance abuse	−0.034	1.421	0.001	0.981	0.967 (0.060–15.654)
Psychotic feature	0.726	1.359	0.286	0.593	2.067 (0.144–29.665)
Suicidal ideation	0.857	1.749	0.240	0.624	2.357 (0.077–72.617)
Constant	4.018	3.441	1.363	0.243	

p < 0.05 was regarded as statistically significant using binomial logistic regression with forced entry

**Table 4:** Binomial logistic regression analysis of factors for divorce among female participants

Variables in the equation	Coeffiecitnet	Standard error	Wald value	p value	Odds ratio (95% conficence interval)
BMI at baseline	−0.399	0.161	6.167	0.013	0.671 (0.49–0.919)
Age at baseline	0.023	0.046	0.257	0.612	1.024 (0.935–1.120)
Age of onset	−0.078	0.054	2.107	0.147	0.925 (0.832–1.028)
Bipolar disorder subcategories (reference; Bipolar I disorder)			1.763	0.414	
Bipolar II disorder	16.597	2545.681	0.000	0.995	1.614*10^7^(0.000–Infinity)
Unclassifiable	18.236	2545.681	0.000	0.994	8.315*10^7^(0.000–Infinity)
Work status at baseline (employed)	−1.361	1.020	1.778	0.182	0.257 (0.035–1.895)
Educational background (reference; Junior college, technical college, or higher)			0.023	0.989	
Special support education school, junior high school	−16.103	5529.838	0.000	0.998	0.000 (0.000–Infinity)
High school, vocational school	−0.131	0.871	0.023	0.880	0.877 (0.159–4.833)
Mood status(reference; Depressive state)			1.228	0.746	
Manic/hypomanic state	−15.843	5301.560	0.000	0.998	0.000 (0.000–Infinity)
Mixed feature	−2.353	2.123	1.228	0.268	0.095 (0.001–6.101)
Remission	−0.092	1.084	0.007	0.933	0.912 (0.109–7.638)
Intelligence quotient (85 or less)	−18.885	6198.986	0.000	0.998	0.000 (0.000-Infinity)
Global assessment of functioning (reference; 81–100)			0.004	0.998	
61–80	−0.061	1.099	0.003	0.956	0.941 (0.109–8.102)
1–60	0.000	1.714	0.000	1.000	1.000 (0.035–28.764)
Mood stabilizers prescription	−0.734	0.871	0.710	0.399	0.480 (0.087–2.647)
Antipsychotics prescription	0.030	0.870	0.001	0.972	1.031 (0.187–5.669)
Antidepressants prescription	0.096	0.901	0.011	0.915	1.101 (0.188–6.438)
Anxiolytics prescription	1.737	0.878	3.910	0.048	5.678 (1.015–31.751)
Hypnotics prescription	0.570	0.873	0.425	0.514	1.768 (0.319–9.793)
Rapid cycler at baseline	−17.698	3840.923	0.000	0.996	0.000 (0.000–Infinity)
Manic symptoms during 1 year before baseline	−0.440	1.284	0.117	0.732	0.644 (0.052–7.975)
Hospitalization during 1 year before baseline	−19.559	7102.789	0.000	0.998	0.000 (0.000–Infinity)
Personality disorder	1.322	1.595	0.687	0.407	3.751 (0.165–85.477)
Developmental disorder	−15.223	6748.552	0.000	0.998	0.000 (0.000–Infinity)
Physical comrbidity	0.554	1.011	0.300	0.584	1.74 (0.240–12.615)
Substance abuse	−15.642	8356.816	0.000	0.999	0.000 (0.000–Infinity)
Psychotic feature	−17.811	5717.615	0.000	0.998	0.000 (0.000–Infinity)
Suicidal ideation	2.067	1.489	1.927	0.165	7.902 (0.427–146.292)
Constant	−10.098	2545.684	0.000	0.997	

p < 0.05 was regarded as statistically significant using binomial logistic regression with forced entry

## Discussion

In this study, we aimed to identify predictors of divorce in Japanese outpatients with bipolar disorder based on observations from psychiatrists at Japanese psychiatric clinics. During the two-year follow-up period, we found that the divorce rate was 2.8%. According to the latest statistics from the Japanese Ministry of Health, Labor and Welfare, the annual divorce rate in Japan was 1.57 per 1,000 population (0.157%) in 2020 [33], so we estimate that the 2-year divorce rate for the general population in Japan is approximately 0.314%.

The key finding of our study is the association between younger age and an increased risk of divorce, which is consistent with previous research [34]. This is consistent with previous research indicating that younger couples may face greater challenges in maintaining a stable relationship due to factors such as financial instability, lack of preparation before marriage, and communication problems [34, 35]. For individuals with bipolar disorder, the combination of these factors with the challenges of managing a psychiatric condition might exacerbate the risk of marital dissolution [8]; however, it should be noted that the average age among those who continued to be married was 54.91 years, while the average age among those who experienced divorce was 46.83 years. Based on these results, it was considered necessary to pay attention to the fact that our study's target population is older than the marriageable age.

The fact that the average age of the divorced group was not particularly young may suggest that cultural and societal factors specific to Japan might influence the relationship between age and divorce risk in this population. For example, in Japan, there is a strong emphasis on valuing family unity, and the social stigma associated with divorce is deeply rooted compared to Western countries [36]. As a result, even in the face of difficulties in their married life, couples may be inclined to endure and stay together [36]. This could potentially delay the decision to divorce until a later age compared to other countries where divorce might be more socially accepted.

In addition, we also found that there was a sex difference in the risk of divorce. The results of this study highlight the importance of considering both similarities and differences in understanding the risk factors for divorce in this bipolar disorder population.

In terms of clinical factors, our findings indicate that men with bipolar I disorder had a higher risk of divorce. Bipolar I disorder is characterized by more severe manic episodes, which may be more disruptive to relationships due to impulsive decision-making, irritability, and an increased risk of engaging in risky behaviors [1]. Additionally, spouses of individuals with bipolar I disorder may experience increased caregiver burden and be more likely to perceive the relationship as unmanageable [1]. The more severe symptoms experienced by individuals with bipolar I disorder can pose greater challenges in managing relationships and may have increased the risk of divorce for men.

Bipolar I disorder is generally associated with more severe symptoms, which include full manic episodes, compared to bipolar II disorder, which involves milder hypomanic episodes [2]. The more severe symptoms experienced by individuals with bipolar I disorder may pose greater challenges in managing relationships and could contribute to a higher risk of divorce in men. On the other hand, it has been suggested that women are more likely to seek social support and emotional connection during times of stress and crisis [37]. Thus, it is possible that women's coping skills helped them maintain relationships regardless of their bipolar disorder subtype. Interestingly, substance use disorders and reduced social functioning, both closely linked to bipolar I disorder [4], did not emerge as statistically significant predictors of divorce. In our study, we do not have information on psychiatric symptoms or family background at the time the decision to divorce was made. Consequently, it is possible that bipolar I disorder, compared to bipolar II disorder, may have contributed to divorce due to a recurrence of mood episodes or the exacerbation of coexisting conditions during the progression of the illness.

For women, a lower BMI at the start of the observation period was a significant risk factor for divorce within the next two years. It is possible that the relationship between BMI and divorce may be mediated by factors such as self-esteem, body image, and perceived attractiveness [38, 39]. Interestingly, while lower BMI was a significant risk factor for divorce among women, it was not a significant factor among men. This sex difference could be attributed to societal expectations and cultural factors that place greater emphasis on physical appearance and attractiveness for women than men [38]. Especially in Japan, the culture of self-repression can cause low self-esteem and social anxiety in women, which are in turn associated with negative body image and a desire to be thin [38]. It can be speculated that this cultural background, which is different from that of Western countries, influences women's weight as a predictor of divorce. Previous studies examining the relationship between marital status and BMI in the Japanese population have shown that BMI does not change with marriage in men, while BMI increases with marriage in women [39]. On the other hand, a study examining marital satisfaction and weight change found that spouses with less satisfied partners were more likely to consider divorce, which in turn reduced weight gain [40]. Therefore, the results of this study suggest that women with bipolar disorder who had inadequate support from their families and experienced anxiety may have chosen to divorce due to dissatisfaction with their relationship with their partners.

In addition, previous research has reported that women experience more depressive episodes [41, 42], rapid cycling and mixed states [43] than men. Considering this, it is possible that in women, appetite loss may occur during depressive episodes, leading to weight loss. Additionally, during depressive episodes, worsening of pessimistic emotions may increase the likelihood of making negative decisions such as divorce. On the other hand, baseline mood status was not statistically significant as a predictor of divorce. Therefore, further research is needed to investigate mood status at the time of divorce, the severity and duration of each episode during the observation period, and their impacts.

It is also possible that weight loss due to comorbid eating disorders with bipolar disorder appears to increase the risk of divorce. Eating disorders are more common in females with bipolar disorder than in males [44]. A similar previous study also found that individuals with comorbid bipolar disorder and eating disorders had more severe bipolar disorder symptoms and were more likely to attempt suicide [45]. Interestingly, eating disorders associated with bipolar disorder have been found to be associated with more bulimia nervosa than anorexia nervosa [44]. Therefore, if we hypothesize that eating disorders affect divorce, it would be clinically useful to stratify and analyze the complication rate of anorexia nervosa. However, our study design did not evaluate the presence or absence of comorbid eating disorders individually, so we could not fully observe the relationship between eating disorders and divorce.

Furthermore, for women, taking anxiolytics emerged as a significant risk factor for divorce. This finding could suggest that the presence of anxiety symptoms or the use of specific medications may contribute to relationship difficulties, such as increased interpersonal conflicts, communication difficulties, or reduced emotional intimacy. A previous study found that anxiety disorders were more prevalent in females with bipolar disorder than in males [46]. Another study indicated that the presence of anxiety disorders was associated with more severe bipolar disorder symptoms and a higher likelihood of hospitalization [47]. Thus, our findings suggest that screening for anxiety disorders in females with bipolar disorder may help identify individuals who require more intensive treatment for family relationships. However, caution is needed in interpreting our findings as it is unknown whether outpatients with bipolar disorder taking anxiolytics in our study were diagnosed with anxiety disorders. Notably, in the treatment of anxiety disorders, antidepressants are generally chosen more often than anxiolytics [48]. Therefore, further investigation is warranted regarding why the prescription of anxiolytic medications at baseline is associated with subsequent divorce.

In American studies, it has been found that women who are divorced have a higher prevalence of generalized anxiety disorder than women who are married [49, 50]. In addition, a Danish cohort study [51] found a correlation between divorce and depression or anxiety disorder regardless of sex, but it was noted that women are more likely to be suspected of having these mood disorders among those who have divorced. Therefore, support focused on women may be effective for anxiety disorders.

There are several limitations to this study. First, the study only targets outpatients attending psychiatric clinics in Japan, which may lead to population bias. Additionally, although this study analyzed predictors of divorce within two years from baseline in patients with bipolar disorder, the mental state and family environment of patients and their partners at the time of deciding to divorce are unknown. This study does not consider the duration of marriage, and the age group of the analysis population is older than the marriageable age, making it uncertain whether the study results apply to relatively younger patients.

In this study, we have focused our analysis solely on bipolar disorder patients. Since we did not establish a control group of healthy individuals, it is uncertain whether the predictors of divorce reported in this study are specific to bipolar disorder patients, and whether the condition's severity contributes to divorce. Additionally, essential details such as divorce circumstances or reasons, mood status, and severity at the time of divorce were not collected; thus, further research is needed to address these aspects.

Furthermore, due to the limited sample size, there were factors with large variations in the analysis results after stratification by sex. Therefore, it was considered that more accurate survey results could be obtained by conducting a large-scale survey targeting a broader age range. Future research should continue to explore factors contributing to divorce risk among individuals with bipolar disorder, with a particular focus on relationship variables, such as partner support and communication skills.

## Conclusions

Our study contributes to understanding the factors predicting divorce among Japanese outpatients with bipolar disorder. Our findings suggest that younger age, lower BMI, bipolar I disorder, and the use of anti-anxiety medications are associated with an increased risk of divorce within the next two years. Consequently, we recognize the importance of establishing a support system for daily life early on, encompassing diet, to ensure family stability. Furthermore, attention to comorbid anxiety disorders, appropriate pharmacotherapy for bipolar I disorder, and confirmation of adherence to treatment are necessary. Recognizing divorce as an issue that impacts and significantly influences the lives of both spouses, we believe that it is essential to communicate the insights gained from this research not only to the patients themselves but also to their family members, providing support from a family perspective.

## Data Availability

The Ethics Committee of the Japanese Association of Neuro-Psychiatric Clinics imposed data-sharing restrictions due to the potential presence of identifiable or sensitive patient information. To request data, please contact the institutional review board of the ethics committee. The contact information for our ethics committee is as follows: The Institutional Review Board of the Ethics Committee of the Japanese Association of Neuro-Psychiatric Clinics; Shibuya-ku, Yoyogi 1-38-2, Tokyo Metropolis, Japan, Postal Code 151–0053, Phone + 81-3-3320-1423.

## Abbreviations

JAPC: The Japanese Association of Neuro-Psychiatric Clinics
MUSUBI: Multicenter treatment SUrvey on BIpolar disorder in Japanese psychiatric clinics
BMI: Body mass index
IQ: Intelligence quotient
GAF: Global Assessment of Functioning
